# The role of gut microbiota in T cell immunity and immune mediated disorders

**DOI:** 10.7150/ijbs.79430

**Published:** 2023-02-13

**Authors:** Ju A Shim, Jeong Ha Ryu, Yuna Jo, Changwan Hong

**Affiliations:** 1 Department of Anatomy, Pusan National University School of Medicine, Yangsan 50612, Republic of Korea; 2 PNU GRAND Convergence Medical Science Education Research Center, Pusan National University School of Medicine, Yangsan 50612, Republic of Korea

**Keywords:** microbiome, T cell, immune system, autoimmune disease, fecal microbiota transplant

## Abstract

Gut microbiota was only considered as a commensal organism that aids in digestion, but recent studies revealed that the microbiome play a critical role in both physiological and pathological immune system. The gut microbiome composition is altered by environmental factors such as diet and hygiene, and the alteration affects immune cells, especially T cells. Advanced genomic techniques in microbiome research defined that specific microbes regulate T cell responses and the pathogenesis of immune-mediated disorders. Here, we review features of specific microbes-T cell crosstalk and relationship between the microbes and immunopathogenesis of diseases including in cancers, autoimmune disorders and allergic inflammations. We also discuss the limitations of current experimental animal models, cutting-edge developments and current challenges to overcome in the field, and the possibility of considering gut microbiome in the development of new drug.

## Introduction

The microbiota, which has been studied for decades, forms a complex network evolving with humans 1-3. The gut microbiota influences the development, homeostasis, and function of the immune system 4, 5.

The gut microbiota interacts to T cells with antigen-specific recognition 6, or signals via Toll-like receptors and Nod-like receptors. These signals mediate cell induction and function, thus ensuring homeostasis in the human immune system 7. The microbiota species has been shown to be associated with the differentiation of T cells such as helper T (Th) 1, Th2, Th17, and regulatory T (Treg) cells 8-10. Also, short-chain fatty acids (SCFAs), metabolites of the gut microbiota, regulate T cell differentiation and activation 11. In addition, germ-free (GF) mice have reduction of Treg cells, the absence of Th17 cells, and imbalance of Th1/Th2 cells, which is skewed into the Th2 response 12. The immune phenotype in GF mice was modulated to Th1 skewed-responses by reconstitution of specific microbe 8. In recent mass cytometry by time-of-flight (CyTOF®) analysis, immune landscape in specific pathogen-free (SPF) mice was significantly distinct from wildling mice which has wildling bacterial microbiome and wildling mice was more similarly phenocopied the immune response of humans than SPF mice 13. Therefore, these studies indicate that gut microbiota can regulate immune landscape and responses including differentiation, homeostasis and development of T cells, and alternation of gut microbiota may be closely linked to the pathogenesis of immune-mediated diseases.

Gut microbiota dysbiosis is caused by a variety of mechanisms, including microbiome imbalance, immune dysregulation, proinflammatory mechanisms, and metabolic activities. Dysbiosis lead to various T cell-related diseases, including rheumatoid arthritis (RA), type 1 and type 2 diabetes, asthma, cardiovascular disease, inflammatory bowel disease (IBD), cancer, liver disease, and psychiatric disorders 14-20.

Despite the importance of understanding microbiome-T cell interactions, most immunology experiments have been performed with limited microbiome composition such as SPF or GF mice. Thus, experimental results using gnotobiotic mouse models in the drug development stage cannot fully represent humans 21. To address these limitations, a fecal microbiota transplant (FMT) mouse model with a gut microbiota similar to that of humans was developed using human or wild mouse feces 22-24. The FMT mouse model has an abundant and more diverse gut microbiome than the current experimental animal models 23. In addition, mice implanted with the wild mouse or human microbiota not only exhibit different degrees of microbial diversity but their systemic immunity is also affected.

Here, we discuss the specific microbes-T cell crosstalk, and distribution of the gut microbiota according to T cell-mediated diseases. In addition, we discuss the need to use the FMT mouse model to overcome the limitations of the current experimental mouse model, and to consider the human gut microbiota in drug discovery.

## Microbiota and T cells

Numerous studies have been conducted on the crosstalk between the gut microbiota and immune cells. Commensal microbiota affects the function, development, and differentiation of T cells to maintain host immune homeostasis.

A recent report that the microbiome acts as an antigen for T cells, specific microbes, such as segmented filamentous bacteria (SFB), contribute to the development of microbiota-specific T cells in the thymus 25. T cells mainly regulate adaptive immune responses and can be divided into CD4^+^ T cells and CD8^+^ T cells. Specific commensal microbiota induces Th cell polarization and cytotoxic CD8^+^ T cell activity. In addition, unconventional T cells including natural killer T (NKT), γδ T, and mucosal-associated invariant T (MAIT) cells recognize microbe groups based on their microbe-derived metabolites.

### CD4^+^ T cells

**Figure 1:**
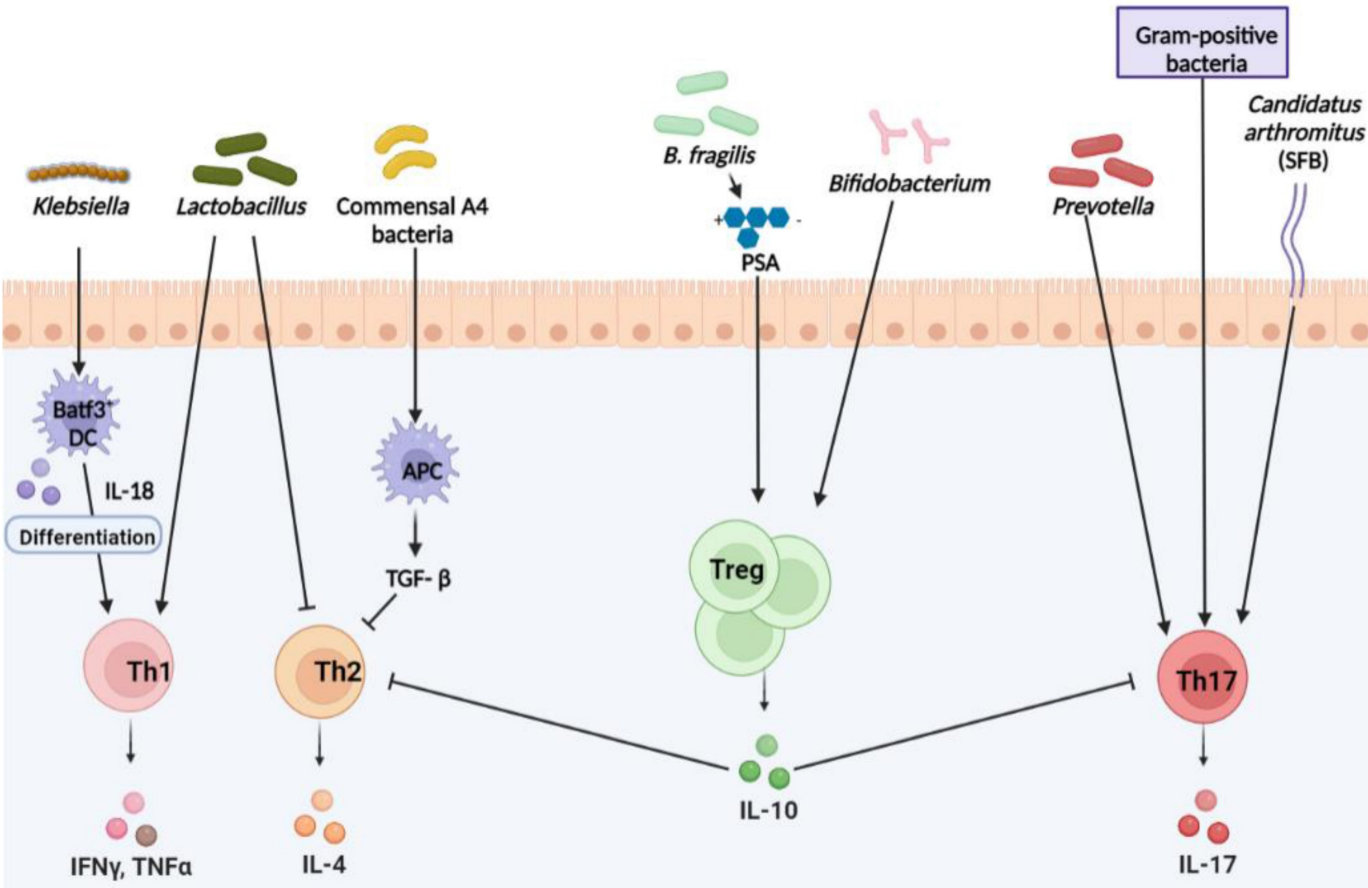
** Microbiota and CD4^+^ T cells.** Microbiota mediates T cell differentiation in homeostatic and pathogenic conditions. *Klebsiella* activates T helper (Th) 1 cell in the intestinal via antigen presenting cells (APCs) to secrete IFNγ and TNFα. *Lactobacillus* activates Th1 cells but inhibits Th2 cells. Symbiotic A4 bacteria of the *Lachnospiraceae* family inhibit the production of IL-4 secreted by Th2 cells by APCs. Th17 cells are activated by* Prevotella*, gram-positive bacteria, and SFB, secreting IL-17 and causing inflammation. *Bacteroides fragilis* (*B. fragilis*) produces PSA, which activates regulatory T (Treg) cells. *Bifidobacterium* also activates Treg cells, which relieve inflammation by inhibiting or balancing Th1, Th2, and Th17 cells. * PSA: Polysaccharide A, a capsular carbohydrate from the commensal gut bacteria *B. fragilis* * Batf3: basic leucine zipper ATF-like transcription factor 3

CD4^+^ T cells differentiate into various Th lineages with different effector functions. Several studies have demonstrated the role of the microbiome in Th cell differentiation and homeostasis. *Klebsiella* (*K.*) genera, such as* K. aeromobilis* and *K. pneumoniae,* induce Th1 cell responses in the gut (Fig. 1). Colonized *Klebsiella* in GF mouse intestines enhances Th1 cell proliferation, leading to Th1 cell augmentation in the intestine 26. Probiotic bacteria have also been shown to modulate Th1 cell activity. Representative probiotic *Lactobacillus* strains are closely related to Th1 cells. *Lactobacillus* (*L.*)* plantarum* 27, 28 and *L. salivarius* 29 enhanced the production of Th1 cytokines, tumor necrosis factor alpha (TNFα), and interferon gamma (IFNγ). *Lactobacillus* strains isolated from fermented foods also upregulated TNFα secretion via macrophage activation*,* simultaneously, the amount of the Th2 cytokine interleukin (IL)-4 decreased 30. Based on these data, the gut microbiota could be involved in both Th1 and Th2 cell activities, and other *Lactobacillus* studies support this hypothesis 28, 31. Mazmanian et al. reported that GF mice appeared more biased toward Th2 cell responses than Th1 cell. However, colonization of GF mice with polysaccharides (PSA) produced by *Bacteroides* (*B.*)* fragilis* corrected the imbalance by skewing Th2 cell to Th1 cell, resulting in decreased IL-4 and increased IFNγ production 8. This indicated that the microbiota could concurrently affect Th1 and Th2 cells. *Klebsiella*-induced Th1 cell differentiation is mediated by basic leucine zipper ATF-like transcription factor 3 (Batf3)-dependent dendritic cells (DCs) and TLR signaling pathway with IL-18 signaling 26. Under homeostatic control, *Klebsiella*-specific Th1 cells can be regulated without induction of severe gut inflammation. Once *Klebsiella* are dominant during dysbiosis, severe gut inflammation can be elicited following induction of Th1 cell differentiation. Indeed, enrichment of *Klebsiella* strains in the fecal of IBD patients than healthy controls has been reported 32.

Th2 cell secreting IL-4, IL-5, and IL-13 significant role in humoral immunity and defense against helminth infections and contribute to chronic inflammatory diseases, such as asthma and allergy 33. *Lactobacillus* strains and *B. fragilis* were found to inhibit Th2 activity by positively influencing Th1 activity 8, 28, 30, 31 (Fig. 1). The influence of the microbiota on Th2 activity appears during the IBD phase. Bamias et al. reported that SAMP1/YitFc mouse models of Crohn's disease (CD) -like ileitis indicated that commensal bacteria induced Th2 response characteristic of the chronic phase of SAMP1/YitFc ileitis, and symptoms also exacerbated 34. Studies have shown that *Lachnospiraceae* strains are Th2 inhibiting microbes. Commensal A4 bacteria, a member of the *Lachnospiraceae* family, induce transforming growth factor-beta (TGF-β) production from DCs, which results in the inhibition of Th2 cell differentiation and activity 35.

Th17 cells produce IL-17, a potent proinflammatory cytokine that causes tissue damage and is involved in the pathogenesis of inflammatory and autoimmune diseases 36. Also, Th17 cells are absent in GF mice and are inducible upon microbial colonization 12 Studies have reported that SFB and gram-positive bacteria induce Th17 cell differentiation (Fig. 1). Atarashi et al. reported that SFB or commensal bacteria-derived ATP activates a specific subset of lamina propria cells, and promotes inducing Th17 cell differentiation 37, 38. In addition, Ivanov et al. reported that SPF mouse treatment with vancomycin, a gram-positive bacterial antibiotic, reduced the frequency of Th17 cells in the small intestine, implicating specific bacteria in Th17 cell differentiation 39. According to Koji and Ivanov, Th17 cell differentiation is determined by gut microbes. Subsequent studies revealed that SFB or '*Candidatus Arthromitus*' is a gram-positive bacterium that promotes Th17 cell differentiation and secretion of several cytokines, such as IL-17 and IL-22 secretions 37, 40-42. Also, *Prevotella* is another bacterium responsible for robust Th17 cell and cytokine secretion in the mouse colon 43. Hang et al. reported that a microbial-produced secondary bile acid negatively affected Th17 cell differentiation. Among 30 different primary and secondary bile acids, 3-oxolithocholic acid blocked Th17 transcription factor RORγt, and Th17 cell differentiation was reduced in SPF mice 44. Although how intestinal Th17 differentiation is induced by specific bacteria was fully undefined, many reports have clearly shown that the microbiome directly and indirectly contributes to Th17 cell differentiation and function. Therefore, Th17 differentiation by the microbiota colonization is linked across diverse autoimmune diseases, such as RA, multiple sclerosis (MS) and IBD, discussed in more detail in the 'Autoimmune diseases' section.

Treg cells play a crucial role in preventing autoimmune diseases through the modulation of immune responses, maintaining immune homeostasis, and providing immune tolerance to both self and non-self-innocuous antigens 45. *B. fragilis* is a well-known microbe capable of robust Treg cell populations by producing PSA. This microbial product leads to the development of Foxp3^+^ Treg cells with IL-10 production 46 (Fig. 1). *Bifidobacterium* strains also affect Treg cells, and it was confirmed that* Bifidobacterium* (*Bi.*)* infantis* and* Bi. bifidum* act to promote Treg cell generation 47, 48. *Lactobacillus* strains are involved in Treg cell differentiation and activity, and *Lacticaseibacillus casei* induces Treg cell development and IL-10 secretion 49. Forsythe et al. reported that *L. reuteri* attenuated allergic airway responses in BALB/c mice 50, and that this microbe promotes Foxp3 expression 51. Thus, *L. reuteri*, affects Treg cell development and the severity of autoimmune diseases. *L. acidophilus* strain L-92 (L-92) showed similar effects in BALB/c mice. Under allergic contact dermatitis (ACD) conditions, oral treatment with L-92 resulted in increased Foxp3, IL-10, and TGF-β expression in mice. They concluded that L-92 mediates ACD by enhancing Treg production and Th1 and Th2 responses 52. Another *Lactobacillus* strain, *L. murinus,* has been shown to regulates RORγt^+^ Treg cells in the small intestine, which attenuates lung inflammation associated with *Mycobacterium tuberculosis* infection 53. Microbial bile acid metabolites are involved in Treg cell differentiation and homeostasis. Song et al. observed that SPF mice fed a nutrient-rich diet produced more primary bile acids than GF mice, and this affected the restoration of RORγt^+^ Treg and Foxp3^+^ Treg cells 54. In contrast, lithocholic acid, a secondary bile acid synthesized by bacteria, isoallolithocholic acid, inhibits Treg cell differentiation 44. Zhang et al. reported that treatment with the penicillin antibiotic, ampicillin, reduced Treg cell proliferation and deregulated the Th1 response to bacterial infection. It can be seen that dysbiosis by antibiotics affects Treg cell production 55. Likewise, microbiota can be altered by various factors, and this change also affects Treg cell generation and activities. Although the mechanism of intestinal Treg cell differentiation by specific commensal bacteria was not clearly defined, intestinal Treg cell colonization is clearly connected to specific bacteria and autoimmune diseases, for example, decreased abundance of *Clostridia* strains in RA and ameliorative effect of *B. fragilis* in IBD, discussed in more detail in the 'Autoimmune diseases' section.

### CD8^+^ T cells

**Figure 2:**
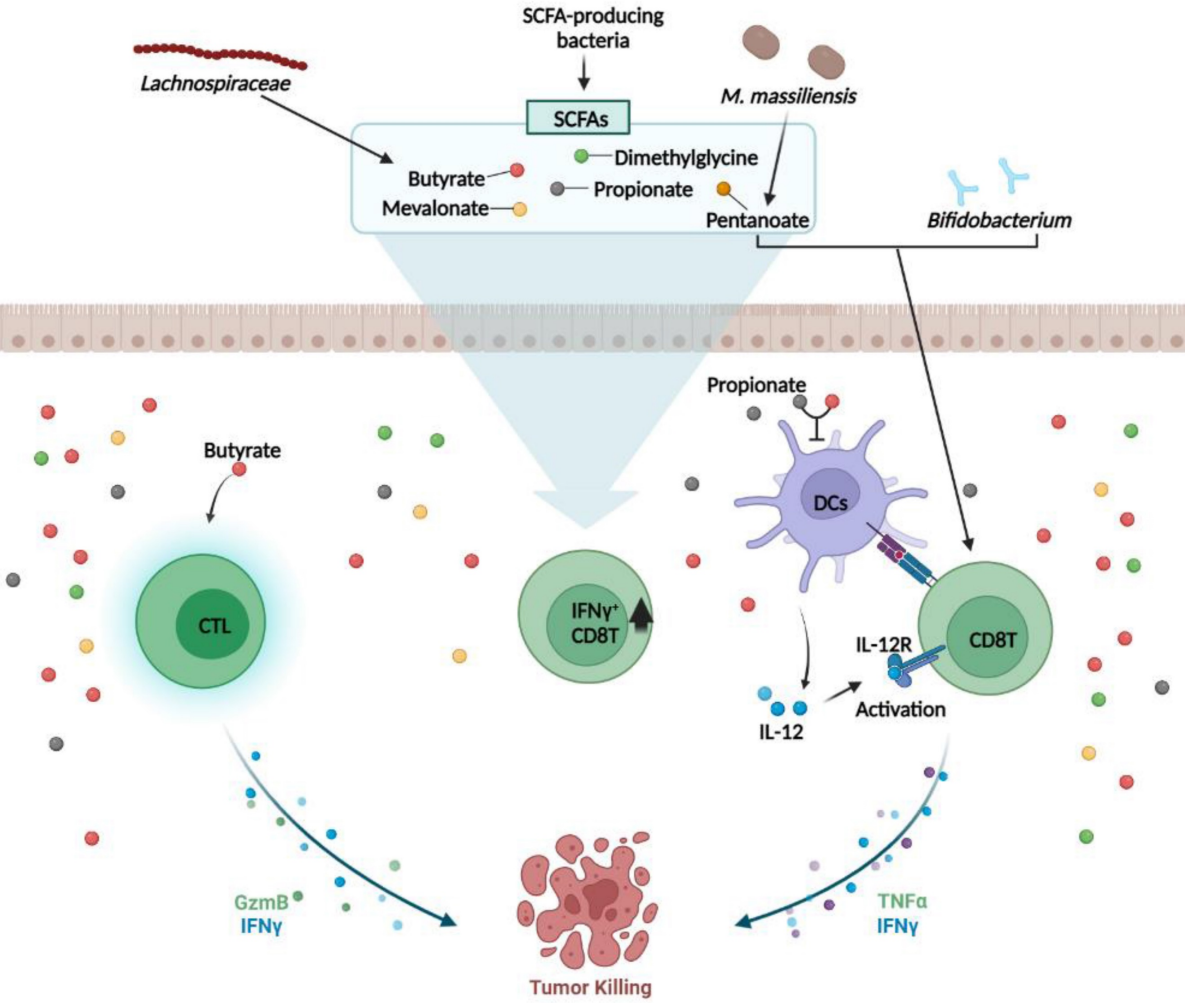
** Microbiota and CD8^+^ T cells**. *Bifidobacterium* and *Mobilicoccus massiliensis* (*M. massiliensis*)-derived pentanoate activates CD8^+^ T cells and increases the secretion of IFNγ and TNFα, thereby enhancing the anti-tumor CD8^+^ T cell response. *Lachnospiraceae* species-derived butyrate and propionate, the two major short-chain fatty acids (SCFAs), decrease APC-induced IL-12 production, thereby inhibiting CD8^+^ T cell activation and IFNγ secretion. In addition, butyrate directly activates cytotoxic T lymphocyte (CTL) cells via other pathways to upregulate the secretion of IFNγ and granzyme B (GzmB). Microbiota-derived SCFAs mediate anti-tumor responses by modulating CD8 T cell responses. SCFAs such as mevalonate and dimethylglycine increase IFNγ^+^CD8 T cells in the intestinal. *TNF: tumor necrosis factor

CD8^+^ T cells are critical for immune defense against intracellular pathogens, including viruses and bacteria, and for tumor surveillance 56. Sivan et al. showed that probiotic species (*Bifidobacteria*) could determine the anti-tumor efficacy of CD8^+^ T cells 57 (Fig. 2). Vétizou et al. showed that particular microbes can influence cytotoxic T lymphocyte antigen-4 (CTLA-4) blockage for cancer immunotherapy. They found that* B. fragilis*,* B. thetaiotaomicron*, and* Burkholderiales* were the major microbes that restored the anti-tumor efficacy of αCTLA-4 treatment 58. Recent studies have demonstrated that microbial byproducts mediate CD8^+^ T cell function. Butyrate and propionate, two major SCFAs, inhibit CD8^+^ T cell activation by controlling IL-12 production by antigen presenting cell (APC) 59. Another study confirmed that butyrate directly increases CD8 T cell activity by upregulating IFNγ and granzyme B expression 60. Luu et al. demonstrated that* Megasphaera* (*M.*)* massiliensis* produced pentanoate promotes effector CD8 T cells activity. In the presence of* M. massiliensis*, they detected higher IFNγ and TNFα expression, and this positively influenced the efficacy of adoptive T cell therapy 61 (Fig. 2). As a result, Microbiota-derived SCFAs modulate CD8 T cell responses during cancer immunotherapy. In contrast, Yang et al. discovered that butyrate derived from* Lachnospiraceae* species inhibited IFNγ secreting CD8 T cells. They elucidated that butyrate restrained the stimulator of the IFN gene (STING) activation in DCs, which is associated with CD8 T cell responses, resulting in lower radiotherapy efficacy 62. Induction of IFNγ^+^CD8 T cells may be associated to specific bacterial strains-derived metabolites, such as mevalonate, dimethylglycine 63 and SCFAs 64, which enter circulation and induce systemic activation of CD8 T cells (Fig. 2). Thus, the microbiota can modulate CD8 T cell function and immunotherapy efficacy.

### Unconventional T cell subsets

#### Natural Killer T cells

**Figure 3:**
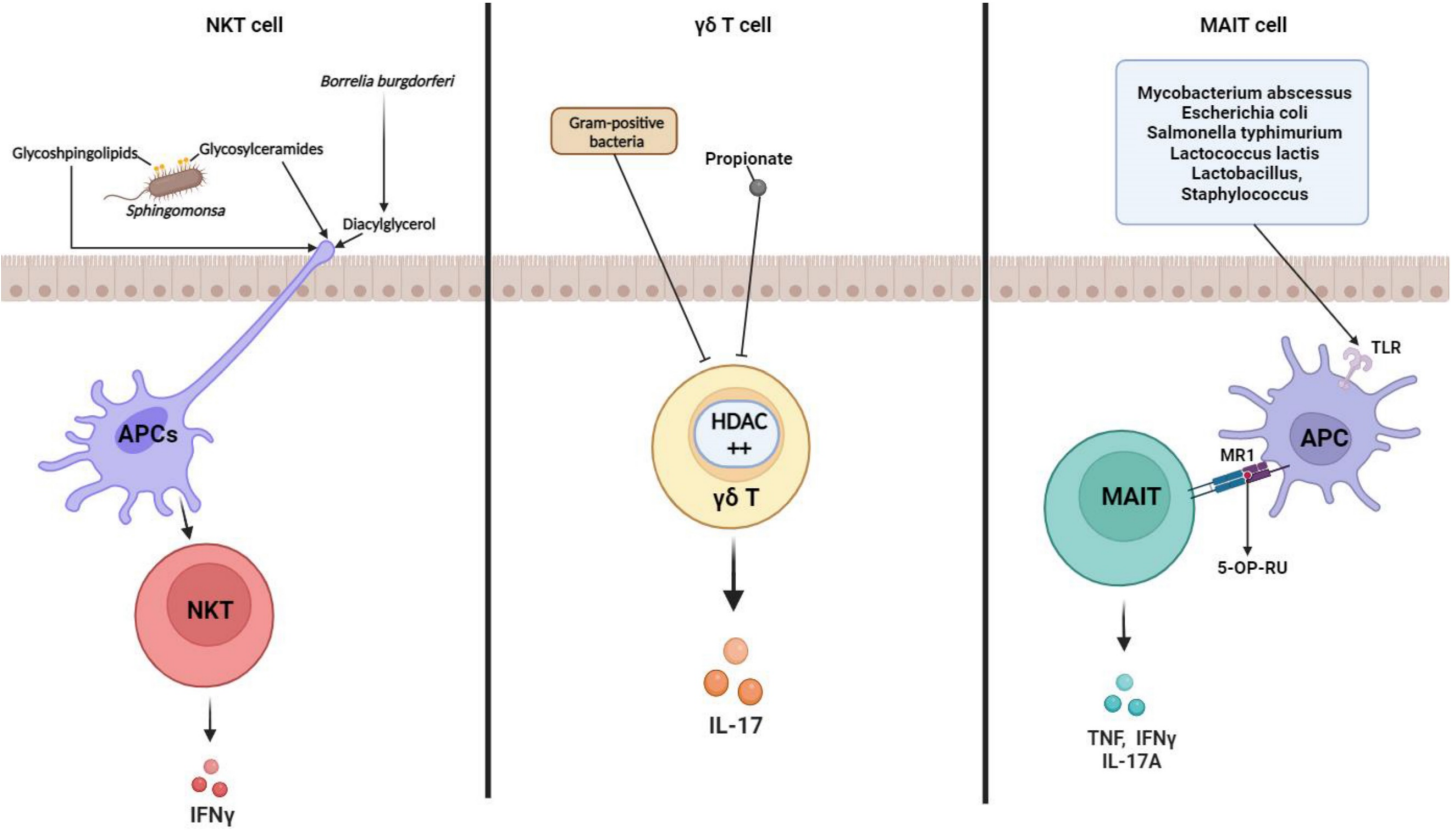
** Microbiota and unconventional T cells.** Modulation of unconventional T cell subset functions by microbe-derived metabolites and their own microbiota. Glycosphingolipids and glycosylceramides from *Sphingomonas* act as microbial antigens to activate NKT cells and secrete IFNγ (left). Gut microbiota represses IL-17 production by cecal γδ T cells. Gram-positive bacteria and short-chain fatty acids (SCFAs; ex. propionate) repress IL-17 producing γδ T cells (middle). Various bacterial antigens (ex.5-OP-RU) presented by APC are presented to MR1 (ligand), bind to the TCR of MAIT cells, activate MAIT cells, and secrete cytokines such as TNF, IFNγ, and IL17A (right). TCR in MAIT cells interacts with MR-1 in APC and is semi-constant. * TCR: T cell receptor; HDAC: Histone deacetylases; TLR: Toll Like Receptors; MR: MHC class I related-1 molecule; 5-OP-RU: MAIT cell ligand

CD1d-restricted NKT cells regulate the broad range of immune responses between the innate and adaptive immune systems. They have the capacity to kill target cells and modulate immune response-secreting cytokines 65, 66. Several studies have revealed that commensal microbiota regulate NKT cells homeostasis. *Sphingomonas* are gram-negative bacteria that are mainly found in the natural environment and are representative NKT cell stimulators. Since glycosphingolipids and glycosylceramides from *Sphingomonas* act as microbial antigens, they stimulate NKT cell activation and IFNγ secretion 67, 68 (Fig. 3). In the immune cells of GF mice, a lower frequency of *i*NKT cells was detected than that in SPF mice, but intragastric administration of *Sphingomonas* increased *i*NKT cell frequency 69. Therefore, *Sphingomonas* exposure affects NKT-cell-modulated diseases. Olszak et al. found that commensal microbiota reduced mucosal *i*NKT cell accumulation. They observed a higher frequency and number of colon *i*NKT cells in GF mice than in that for SPF mice, implicating IBD and allergic asthma morbidity 70. Sphingolipid produced by *B. fragilis* regulates homeostasis with suppression of NKT cell proliferation in neonatal mice 71, indicating that NKT cell-microbiota networking is critical in maintaining of balance between protective responses and excessive inflammation. CXCL16 expression is mediated by microbial bile acids, which affect hepatic NKT cell accumulation. *Clostridium* species modified bile acids promote the CXCL16 level in liver sinusoidal endothelial cells. Moreover, recruiting hepatic NKT cells has shown impressive anti-tumor responses against EL4 lymphoma tumors 72. According to these findings, gut microbiota significantly affects NKT cells and mediates several diseases.

#### γδ T cells

γδ T cells are a small population of T cells that promote inflammatory responses and are particularly important for initial inflammatory and immune responses 69. Similar to other T cell subsets, γδ T cells are also influenced by the microbiome. Under SPF conditions, microbes activate interleukin-1 receptor 1 (IL-1R1) expression on γδ T cells in the small intestine, thereby maintaining IL-17 production 73. Li et al. showed that IL-17A-producing γδ T (γδ T17) cell homeostasis is mediated by the commensal microbiota in the liver. Commensal microbiota affects γδ T17 cell activation and IL-17 cytokine secretion, which accelerates non-alcoholic fatty liver disease (NAFLD) progression 74. Microbial byproducts also downregulated γδ T17 cell activity. Vancomycin treatment increased γδ T17 cells in conventional mice, indicating that gram-positive bacteria repress γδ T cell function. Dupraz et al. confirmed that propionate is the main factor modulating IL-17 secretion by γδ T cells 75 (Fig. 3). Another study showed that commensal microbiota is associated with γδ T cell expansion in the mouse lung, which can promote particulate matter-induced neutrophilia 76. As previously reported, γδ T cells that recognize lipids and phosphoantigens, as intermediates produced by microbiota via metabolic pathway, undergo various immune responses and epigenetic modification. This modification is indirectly induced through microbiota-epithelial cell interaction 77. Although the interrelationship between intestinal γδ T cell and specific commensal bacteria was not clearly defined, localization and motility of γδ T cells are microbiota-dependently regulated in the epithelial layer 78 which is critical in protective immunosurveillance of the gut surface, indicating that commensal microbiota play an important role in the protective function of γδ T cells in diseases.

#### Mucosal-associated invariant T cells

MAIT cells are MHC Ib-restricted innate-like lymphocytes and play a critical role in mucosal homeostasis. Interest in MAIT cell-microbiota interaction has increased since some studies have reported that MAIT cells can recognize bacteria and protect against microbial infection 79, 80. Koay et al. showed that the number of MAIT cells in GF mice was noticeably lower than that in SPF mice. They found that the MAIT cell development in the thymus is impaired as the microbiome does not exist in GF mice 81. Subsequent studies corroborated the significance of the microbiome on MAIT cell development and generation 72, 82. Moreover, MAIT cell has an anti-microbial function, and some microbiome infections trigger MAIT cell function enhancement. For example, *Francisella tularensis* 80, *Mycobacteroides abscessus*, *Escherichia* (*E.*)* coli* 83, *Salmonella typhimurium* 84, and *Lactococcus lactis* 85 act as MAIT cell activators. Activation of MAIT cells recognizes various bacterial antigens (eg, 5-OP-RU) presented by MHC class 1b protein MR1 and secretes cytokines such as TNF, IFNγ, and IL-17A (Fig. 3). However, the use of mouse models for MAIT cell studies is much more limited than the use of human MAIT cells. SPF and GF mice lack microbiome complexity and richness, and this microbial environment is not sufficient to develop mouse models for MAIT cell studies. Indeed, MAIT cells are found in human blood and peripheral tissues, including the liver and gut lamina propria 86, 87, and their frequency is higher than that in mice 88. Furthermore, microbiome comparisons between laboratory mice and humans are strikingly different 89; these factors may influence several aspects of host immune system and diseases.

## T cell-mediated inflammatory disease and microbiota

**Figure 4:**
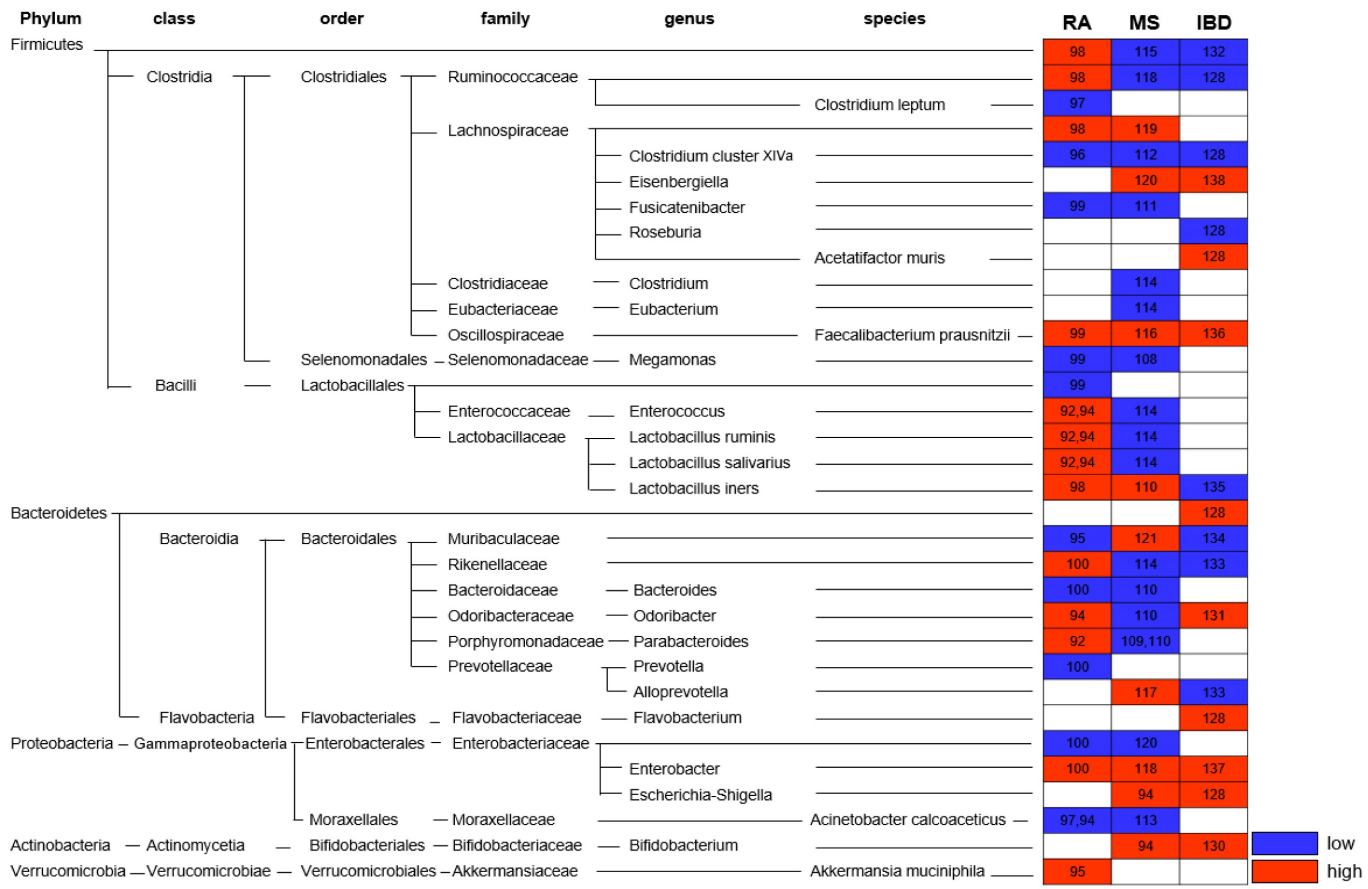
** Alteration of gut microbiota in autoimmune disease.** Altered abundance of the gut microbiota in patients with Rheumatoid arthritis (RA), Multiple sclerosis (MS), and Inflammatory bowel disease (IBD) is indicated. * Red: high; blue: low; number: reference

The etiology of autoimmune diseases can mainly be explained by genetic mutations and environmental factors 90, 91. In particular, individual genetic susceptibility to diseases and subsequent environmental triggers cause changes in the composition of symbiotic microorganisms that interact with the immune system. Recent studies have highlighted the importance of microorganisms in autoimmune diseases such as RA 92, experimental autoimmune encephalomyelitis (EAE) 93, asthma, and IBD 94. In this context, several reports have identified that intestinal microbes and their metabolites regulate T cell differentiation and function 63, 95. In addition, disease progression and severity are affected by the composition of an individual's microbiota 94, 96 (Fig. 4).

### Autoimmune diseases

#### Rheumatoid arthritis

RA is a systemic chronic inflammatory disease that mainly affects the joints and is known to be an autoimmune disease accompanied by bone and cartilage destruction. Although the pathogenesis remains unclear, the microbiome, along with genetic and environmental factors, is also involved in the pathogenesis of RA. Numerous studies have investigated the gut microbiome abundance in patients with RA (Fig. 4). Among them, the abundance of *Firmicutes, Ruminococcaceae, Bacteroides, Prevotella, Lactobacillus* group*, and Faecalibacterium* (*F.*)* prausnitzii* increased, whereas *that of Rikenellaceae, Alloprevotella, Bifidobacterium*, *Clostridium* (*C.*)* cluster XIVa*, *Enterobacter, Enterococcus, Fusicatenibacter, Megamonas, Odoribacter*, and* C. leptum* decreased 97-105. Scher et al. found that in the gut of patients with recent-onset RA, an increased abundance of *Prevotella* (*P.*)* copri* and *Bacteroides* and decreased abundance of *C. leptum* was observed 98.

In the RA mouse model, collagen-induced arthritis (CIA), the Lactobacillaceae lineage was significantly more abundant. The Lactobacillus genus was significantly more abundant after collagen-mediated induction in CIA-sensitive mice than in CIA-resistant mice 92, 106. Ivanov et al. found that Candidatus Arthromitus or SFB colonization induces Th17 cells, rapid onset autoimmune arthritis, and exacerbates CIA 41. Conversely, commensal bacteria-derived butyrate (C. cluster XIVa, including Lachnospiraceae, which are major butyrate producers) suppresses the development or improves symptoms of autoimmune arthritis 107. Numerous studies have shown that SFB and prevotella, which are known to promote the secretion of Th17 cells and inflammatory cytokines, are greatly increased in autoimmune RA. Thus, the differential abundance of the gut microbiota suggests that it may orchestrate the development and symptoms of autoimmune arthritis through a Th cell-mediated pathway.

#### Multiple sclerosis

MS is an autoimmune and chronic inflammatory demyelinating disease of the central nervous system (CNS) caused by proinflammatory Th1 and Th17 cells 108. Although Th17 cells have been reported to have a significant impact on MS pathogenesis, it has been suggested that microbiota alterations may also be factors in MS initiation and severity 99, 109, 110. According to Koji and Ivanov, recruitment and activation of myelin-specific CD4^+^ T cells from immune processes depends on the availability of the target commensal gut microbiota 109. Lee et al. suggested that GF mice harboring only SFB develop EAE, as colonization with SFB induces Th17 (IL-17) in the CNS, and that gut bacteria may influence neuroinflammation 111. Additionally, SFB affects the disease by increasing cytokine production by Th1 (IFNγ) and Th17 (IL-17) cells in EAE (mouse model of MS) 111-113. In patients with MS, *Akkermansia* (*Ak.*)* muciniphila* and *Acinetobacter (Ac.) calcoaceticus* both increased (induced proinflammatory responses), but *Parabacteroides distasonis* was reduced (stimulated anti-inflammatory IL-10-expressing) 99. In addition, the abundance of *Bacteroidetes, Lachnospiraceae, Rikenellaceae, Eisenbergiella, Escherichia-Shigella, F. prausnitzii, and Flavobacterium* increased in patients with MS, whereas *Firmicutes, Ruminococcaceae, Bacteroides, Bifidobacterium, Clostridium, L. salivarius, L. iners, L. ruminis, Megamonas, Odoribacter, Parabacteroides*, and *Prevotella* decreased 99, 114-128 (Fig. 4). These results are similar to those of EAE. EAE onset and severity showed differences based on the composition of the microbiota community. At the onset of EAE, *Acetatifactor* (*A.*)* muris, C. leptum, Turicibacter sanguinis, C. cluster XlVa,* and* Erysipelotrichaceae* families increased*,* whereas *Lactobacillus* decreased 129. In addition, *Bifidobacterium, Prevotella*, and *Lactobacillu*s were negatively correlated with EAE severity, whereas *Anaeroplasma, Rikenellaceae*, and *Clostridium* were positively correlated with disease severity 119, 130. Oral administration of *L. murinus* and *L. reuteri* 119, 131 is effective in reducing Th1/Th17 cells and related cytokines IFNγ/IL-17 and in alleviating and preventing EAE symptoms 109, 110, 132. Altogether, data from MS patients and the murine EAE model found the composition of microbiota correlated with disease onset and clinical severity. Since the immune responses are activated or suppressed depending on the type of gut bacteria, the immunoregulatory effect on MS patients can be applied differently. Therefore, the diverse gut microbiota is expected to provide new preventive and therapeutic approaches targeting autoimmune response in patients with MS.

#### Inflammatory bowel disease

IBD is an autoimmune disease that occurs in the digestive tract owing to chronic inflammation and is divided into CD and ulcerative colitis (UC) 133. Chronic inflammation leads to the production of reactive oxygen species (ROS), which impair DNA. It also controls proinflammatory mediators that promote cellular survival and growth. Consequently, patients with IBD have an increased risk of developing CRC 94. In IBD patients, *Enterobacteriaceae*, *Muribaculaceae*, and *A. muris*, which are commensal proinflammatory bacterial taxa, were overgrown, and the* Firmicutes* family, especially the* Clostridia* class and *Roseburia,* were decreased 134. Numerous studies have shown that the intestinal microbial abundance in IBD patients of *Eisenbergiella, Escherichia-Shigella, F. prausnitzii, Parabacteroides, Ac. calcoaceticus,* and* Ak. muciniphila* were higher*,* whereas *Bacteroidetes, Rikenellaceae, Ruminococcaceae, C. cluster XIVa*, and *Flavovobacterium* showed low abundance 134-144 (Fig. 4). A higher frequency of cells was observed in the gut microbiome of the IBD mouse model compared to mice colonized with healthy donor microbiotas 145. In addition, SPF mice develop colitis with conventional microbiota but not under GF conditions 146. The administration of *Clostridium* has been reported to prevent inflammation by increasing induced iTreg cells that secrete TGF-β and IL-10 10. In addition, *Lactobacillus* (especially *L. murinus*) induces Treg cell expansion by secreting IL-10 and TGF-β in colonic DCs and macrophages, and *B. fragilis* also relieves inflammation by inducing Treg cells 133, 147, 148. IBD is influenced by the composition of the gut microbiota; therapeutic supplementation with probiotics, prebiotics, synbiotics, and FMT is also being investigated to suppress inflammation 149-151. Therefore, it is expected that specific bacterial strains such as *Clostridium* and *B. fragilis* that induce Treg cells in the intestinal environment can be a novel treatment targeting IBD patients with intestinal bacterial dysbiosis.

### Allergic airway inflammation (AAI) and Asthma

Asthma is a chronic airway inflammation disease characterized by airway hyper-responsiveness (AHR), airflow obstruction, airway inflammation, and airway remodeling 152. In addition, eosinophil infiltration and higher concentrations of Th2 cytokines (IL-4, IL-5, and IL-13) and immunoglobulin E (IgE) were detected in the serum and bronchoalveolar lavage fluid of patients with asthma 152, 153. According to current research, maternal or pediatric intestinal microbial clusters affect the immune system and are involved in allergic diseases, such as asthma. Herbst et al. observed that the total number of infiltrating lymphocytes and eosinophils was elevated in the airways of allergic GF mice compared to that in control SPF mice 154. Oral treatment with live *L. reuteri* in a mouse model of allergic airway inflammation could attenuate the symptoms of an asthmatic response by inducing Treg cell production and increasing IL-10 secretion. This microbe also reduces the secretion of inflammatory cytokines, such as monocyte chemoattractant protein1 (MCP1), IL-5, and IL-13 including T lymphocytes and macrophages 50. In addition, robust *L. murinus* Treg cells in the mesenteric lymph nodes and lung and decreased proinflammatory cytokine secretion are related to AAI severity. These data demonstrate that AAI and asthma can also be attenuated by strain-specific probiotics, such as in patients with RA and MS. Antibiotics are known to induce changes in the intestinal microbiota, and among them, azithromycin is reported to alter the gut microbiome and attenuate airway inflammation in allergic asthma 155. In summary, altered gut microbiome may influence the onset and severity of asthma or disease with AAI. In addition, it will provide potential new biomarkers and suggest the possibility of more targeted therapies for autoimmune diseases by regulating the gut bacteria strains.

## Cancers and microbiota

Interactions between the gut microbiome and immune system are thought to influence cancer immune surveillance. The human immune system plays an important role in tumor suppression, as tumor cells express immune checkpoints, such as programmed death (PD) 1/PD-ligand 1 and CTLA-4, to avoid the immune system response and suppress anti-tumor immunity 156, 157. In addition, PD-1 and CTLA-4 expression increased by CD8^+^ tumor-infiltrating T lymphocytes in patients with pancreatic ductal adenocarcinoma (PDAC) 158, 159. Blocked CTLA-4 induces a dramatic decrease *in Bacteroidales* and *Burkholderiales*, with a relative increase in *Clostridiales* 58. Several studies have reported that PD-1 blockade in cancer patients is associated with gut microbiota, including *Akkermansia, Bifidobacterium*, and *Faecalibacterium* 57, 160-162. In addition, cancers, such as CRC and PDAC, may be caused by dysbiosis of the gut microbiome. Numerous studies have analyzed and compared the gut microbiome abundance of different cancer patients and healthy donors. When the gut microbiota was analyzed in patients with various types of cancers such as PDAC, CRC, breast cancer, ovarian cancer, and cervical cancer,* Proteobacteria*, *Firmicutes* phyla and *Actinobacteria* abundance was very high compared to that in a healthy donor 163-166. Conversely, *Bacteroidetes* are more abundant in healthy donors than in cancer patients with PDAC, CRC, ovarian cancer, or breast cancer 167. Dysbiosis in patients with CRC causes increased *Ak. muciniphila, E. coli, P. copri, Alistipes putredinis, Ruminococcus torques*, and* Prevotella* 94, 168. Microbiota-produced SCFA, such as acetate, butyrate, and propionate, have great effects on disease prevention and health promotion. Butyrate is also known to play an important role in cancer prevention 169. In particular, fecal samples of patients with PDAC are depleted in the phylum *Firmicutes*, which includes beneficial bacteria including *F. prausnitzii*, *Eubacterium rectale*, and *Roseburia intestinalis* 170. Butyrate secreted by *Firmicutes* suppresses histone deacetylase activity and downregulates proinflammatory cytokines in intestinal epithelial cells and immune cells to alleviate CRC symptoms 171, 172. Zhang et al. reported that as a result of analyzing the intestinal microflora in lung cancer, lower levels of *Kluyvera*, *Escherichia-Shigella*, *Dialister*, *Faecalibacterium*, and *Enterobacter* were found in patients with lung cancer, whereas *Veillonella*, *Fusobacterium, and Bacteroides* were significantly higher than in healthy individuals 173. In breast cancer patients, the anti-tumor effect was higher in patients with a high abundance of specific gut microbiomes, such as *Ak. muciniphila, Bi. longem, Collinsella aerofaciens,* and *F. prausnitizi* 174. Cheng et al. reported that the gut microbiota in patients with ovarian cancer can negatively regulate estrogen levels 175. Taken together, comparing the abundance of gut microbiota could be a useful biomarker for certain cancer patients and that the gut microbiome influences the occurrence, development, treatment, and prognosis of cancers; it will also provide new strategies for the prevention, diagnosis, and treatment of cancers.

## Study of wildling or humanized mice by fecal microbiota transfer and its necessity

**Figure 5:**
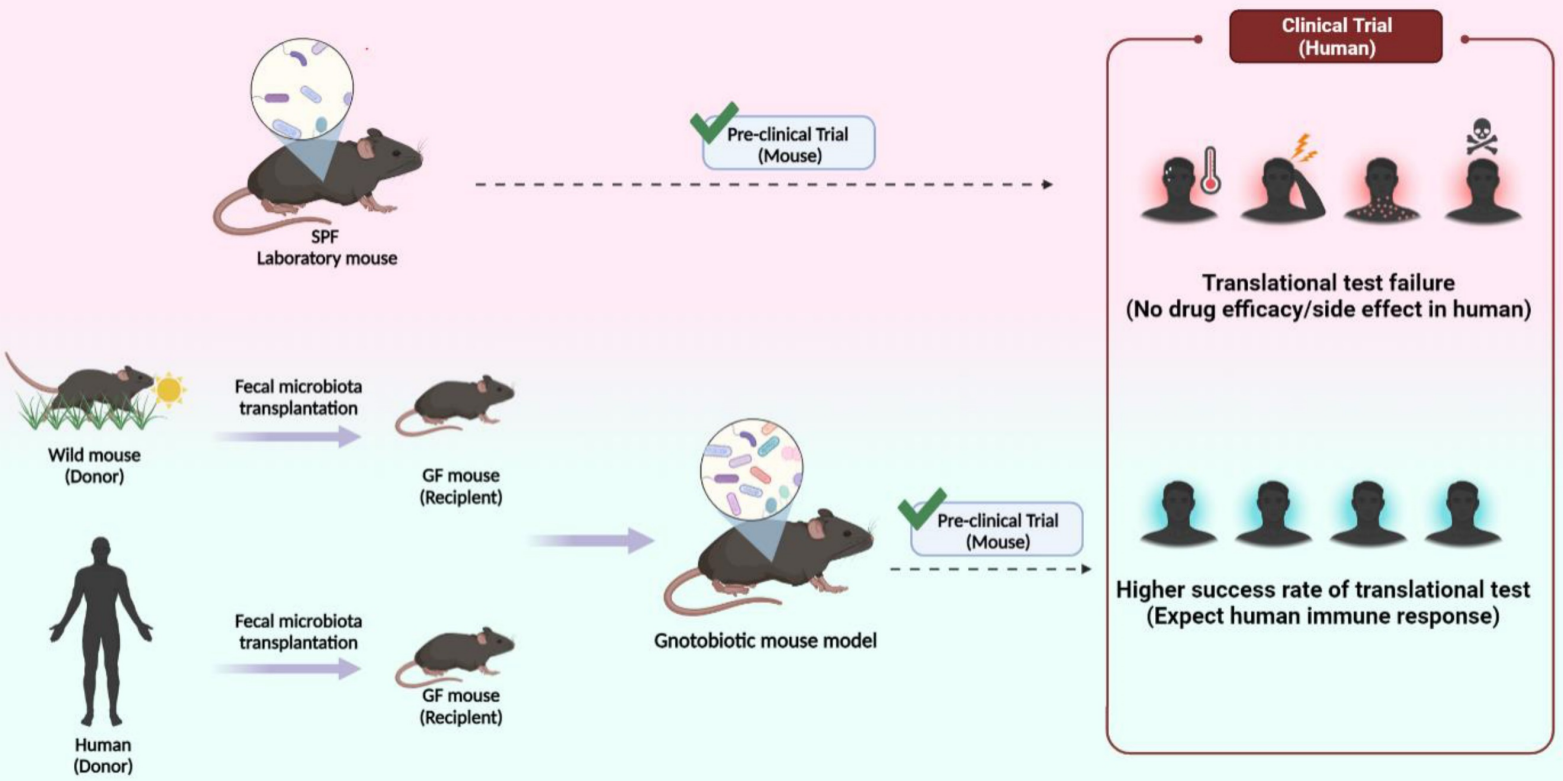
** The necessity for laboratory mouse expressing wild and human microbiome.** SPF experimental mouse currently used a low success rate due to translational failure in the clinical stage despite success in preclinical tests (top). A humanized or wildling mouse model in which feces of the human and wild mouse are transplanted is predicted to show high translational test success rate in clinical trials after successful preclinical tests (bottom).

As a representative experimental animal, the mouse is essential for immunologists and biomedical research because it has more than 90% genetic similarity to humans 176 as well as advantages such as its small size, low cost, and ease of handling. SPF and GF mice with a limited microbiome managed in an ultra-hygienic system to prevent pathogen invasion in the 1960s 177 were used. Ironically, the limited microbiota in laboratory mice is a growing concern in human immunology and clinical research. Unlike laboratory mice, humans have been exposed to external pathogens from birth, which naturally leads to a higher microbiota complexity and abundance in humans than in laboratory mice 89. Furthermore, in terms of the immune system, the microbial challenge from natural habitats matures the human immune system, whereas laboratory mice have an immature immune system similar to that of neonates. According to a study by Beura et al., laboratory mice lacked memory CD8^+^ T cell subsets that experienced protection against pathogen invasion 178. This difference has limited the translational research value from laboratory mice to humans. Especially in the drug development and testing stages, preclinical testing using laboratory mouse model does not always show the same value as clinical testing in humans. The failure rate of translational tests is approximately 90%, mainly because drug candidate toxicity cannot be predicted at the clinical stage 179. Therefore, to increase the success rate of clinical trials, preclinical trials must be reinforced. Therefore, immunologists have proposed the use of wild mice. As wild mice live in natural habitats similar to humans, using wild mice could provide the same environmental conditions as laboratory mice in terms of gut microbiota and immune system aspects. Recent studies have supported the importance of investigating wild mice. Rosshart et al. captured wild mice from Maryland, USA, and compared the gut microbiota with SPF mice. Interestingly, the gut microbiota composition differed between the groups. Relatively higher levels of *Proteobacteria* and *Bacteroidetes* were detected in wild mice, whereas *Firmicutes* levels were lower than in laboratory mice 22. Ericsson et al. also consistently demonstrated that the wild mice microbiota differed from laboratory mice 180. In addition, wild mice showed distinct immune system differences compared with laboratory mice. Wild mice had higher proportions of effector and memory T cells and higher cytokine production 178, 181. Therefore, utilizing the wild mice-microbiome that influences immunological properties could be an option to improve the value of preclinical findings. Recently, wild mouse-based studies have been conducted using various wild microbiota-transplantation methods. It has been reported that the rewilded laboratory mouse via wild microbiota colonization showed similar microbial community 13, 22 and immune system fitness 13, 178, 182-184 and enhanced disease resistance 22. Additionally, mice implanted with human microbiota have been studied for decades to determine their roles in health and disease. Burz et al. reported that when microbiota from a patient with non-alcoholic fatty liver disease (NAFLD) and healthy individuals were transplanted into mice, they displayed a microbiome composition similar to that of a human donor. When a high-fructose, high-fat diet was administered to mice transplanted with intestinal microbes from patients with a fatty liver, NAFLD characteristics, including increased body weight, steatosis, and plasma cholesterol, were observed. These results suggest that gut bacteria play a role in the development of obesity and steatosis and that targeting the gut microbiota could be a preventive or therapeutic strategy for managing NAFLD 24. Another study identified a group of gut microbes that either promoted or inhibited tumorigenesis after transplantation of fecal samples from patients with CRC into GF mice. Baxter et al. suggested that a better understanding of the gut microbiota could lead to the development of prebiotic or probiotic therapies to prevent or delay the development and progression of CRC 185. Britton et al. suggested that the fecal microbiota of humans with IBD alters intestinal CD4^+^ T cell homeostasis in mice and induces more Th2 and Th17 cells. In addition, it has been reported that mice colonized with IBD microbiota in a colitis model suffer from more severe diseases 145. Based on current studies, fecal transfer of microbiota can be utilized to alter the laboratory mouse microbiome community and immune fitness; this process can facilitate the prediction of human immune responses. Therefore, FMT models such as the wildling or humanized model by fecal transfer would enhance the reliability of preclinical tests by providing accurate data in drug efficacy tests (Fig. 5). This would lead to the holistic development of the pharmaceutical industry.

## Conclusion

This review discusses the relationship between gut microbiota and the immune system. In recent decades, significant advances have been made in immunology and microbiology using microbiota and germ-free animals and next-generation sequencing. Many such studies have revealed the correlation between gut microbiota and the immune system. Based on this, we have discussed the role of gut microbiome in activating T cells and the development and promotion of autoimmune diseases and various cancers. FMT mouse models of Human and wild mice are expected to increase the success rate of preclinical studies. Furthermore, it will be possible to overcome the limitations of using GF or SPF mice by gut microbiome research which will open new avenues for disease treatment and prevention based on the distribution of the gut microbiome.
